# The Buffalo General Hospital

**Published:** 1875-05

**Authors:** 


### The Buffalo General Hospital.

The annual report of this institution for the year 1874 is before us, and its contents demand our attention. The Trustees in their report pay a deserved tribute to the Ladies' Hospital Association, and express the wish that the plan of uniting the Executive Committee of the Ladies' Hopital Association and their Board may be continued in operation. The efforts of the ladies have been quite successful in relieving the Trustees of a large share of the direct care of the Hospital, and through their efficient aid a considerable amount of the necessary funds of the institution have been raised.

Considerable interest has been manifested in procuring a permanent endowment, and we understand that the move has met with considerable encouragement. There is now belonging to this fund paid in the sum of $26,000, of this $5,000 was contributed by a charitable lady for the endowment of free beds. There are also bequests amounting to $16,000 not yet paid in.

The report of the Ladies' Hospital Association gives some idea of the work which they undertake, but can convey no correct idea of its magnitude to those unacquainted with the details. A considerable sum of money is contributed by the various churches, but there probably remains a number who never contribute from lack of opportunity, to meet this we think that the plan which has been put in operation in Boston of having one Sunday of the year designated as Hospital Sunday, on which day a general collection could be taken up in all the churches, might be made to work. This plan we suggested in August last, but do not know that its merits have been canvassed by the Ladies' Association.

The report of the Superintendent conveys some interesting intelligence concerning the expenses and general conduct of the Hospital. It closes with a tribute to the faithful attendance of the Resident Physician, who, we can say from personal knowledge has proved himself commendably attentive to his duties.

Medically the report does not contain any information, but we may hope for an improvement in this direction as the Hospital grows. The situation of the Hospital is an excellent one, and the building is very well adapted to its purposes. The wards are high and airy and the hygienic conditions good.

